# Effects of Sleeve Gastrectomy on Blood Pressure Reduction in Diet-Induced Obese Hypertensive Rats: A Potential Role of Prouroguanylin

**DOI:** 10.3390/nu17223581

**Published:** 2025-11-16

**Authors:** Naoki Matsuda, Yuichi Yoshida, Koro Gotoh, Satoshi Nagai, Ryo Kurimoto, Kentaro Sada, Takaaki Noguchi, Miho Suzuki, Shotaro Miyamoto, Yoshinori Ozeki, Takashi Ozaki, Akiko Kudo, Takeshi Nakata, Akihiro Fukuda, Takayuki Masaki, Hirotaka Shibata

**Affiliations:** 1 Department of Endocrinology, Metabolism, Rheumatology and Nephrology, Faculty of Medicine, Oita University, Yufu 879-5593, Japan; matsuda20656@oita-u.ac.jp (N.M.); y-yoshida@oita-u.ac.jp (Y.Y.); n-satoshi@oita-u.ac.jp (S.N.); kurimotoryo@oita-u.ac.jp (R.K.); sadaken@oita-u.ac.jp (K.S.); noguchitakaaki@oita-u.ac.jp (T.N.); m-suzuki@oita-u.ac.jp (M.S.); shoutarou1029@oita-u.ac.jp (S.M.); ozeki23@oita-u.ac.jp (Y.O.); t-ozaki@oita-u.ac.jp (T.O.); kudou3@oita-u.ac.jp (A.K.); akifukuda@oita-u.ac.jp (A.F.); 2 Research Center for Global and Local Infectious Diseases, Oita University, Yufu 879-5593, Japan; 3 Faculty of Welfare and Health Sciences, Oita University, Oita 870-1192, Japan; nakata@oita-u.ac.jp; 4 Geriatric Nursing, Department of Nursing, Faculty of Medicine, Oita University, Yufu 879-5593, Japan; masaki@oita-u.ac.jp

**Keywords:** hypertension, natriuresis, obesity, sleeve gastrectomy, prouroguanylin

## Abstract

**Background/Objectives:** Sleeve gastrectomy (SG) is the most commonly performed bariatric surgery worldwide. It results in significant weight loss and improves metabolic disorders such as hypertension. Weight loss is thought to be the main factor contributing to blood pressure (BP) reduction after SG. Small-intestinal hormones may also mediate the antihypertensive effects of SG. We aimed to investigate the mechanisms underlying the antihypertensive effects of SG through small-intestinal hormones independently of weight loss. **Methods:** This study involved male Sprague–Dawley rats that underwent a sham operation or SG, followed by a dietary intervention involving a standard diet, a high-fat and high-salt diet, or pair-feeding with SG. **Results:** Three weeks postoperatively, SG significantly reduced systolic blood pressure (SBP) and increased urinary sodium excretion. RNA sequencing of the small intestine revealed upregulation of the gene encoding prouroguanylin (proUGN). proUGN is a small-intestinal hormone that inhibits renal sodium reabsorption by converting sodium/hydrogen ion exchanger type 3 (NHE3) in the proximal tubules into the inactive phosphorylated form at Ser552 (pS552-NHE3). Furthermore, SG significantly increased proUGN levels in the ileum and plasma, as well as the levels of pS552-NHE3 in the renal cortex. The administration of exogenous uroguanylin, which is converted from proUGN, resulted in increased renal pS552-NHE3, increased urinary sodium excretion, and decreased SBP without body weight reduction. These effects were similar to those observed with SG. **Conclusions:** SG increases proUGN secretion from the small intestine, leading to increased blood concentration. This inhibits NHE3 activity in the proximal tubules, promotes natriuresis and reduces BP.

## 1. Introduction

Obesity is a major global health problem and a risk factor for metabolic abnormalities, including hypertension, diabetes, and dyslipidemia [1,2,3]. In particular, obesity-associated hypertension increases the incidence of cardiovascular events and worsens long-term prognosis; therefore, effective prevention and treatment are clinically important [4,5,6]. Excess accumulation of visceral fat induces chronic inflammation and insulin resistance via an imbalance in adipocytokines (e.g., leptin) [7]. This imbalance activates the sympathetic nervous system, thereby increasing the heart rate and systemic vascular resistance, as well as promoting renal sodium reabsorption and renin secretion. In addition, leptin directly stimulates adrenal zona glomerulosa cells to upregulate CYP11B2 and increase aldosterone synthesis [8]. These mechanisms ultimately increase blood pressure (BP) [9].

Sleeve gastrectomy (SG) is the most commonly performed type of bariatric surgical procedure [10]. SG induces substantial and long-lasting weight loss and improves metabolic disorders such as hypertension and type 2 diabetes [11,12,13,14]. Meta-analyses of randomized controlled trials have demonstrated that SG significantly lowers BP both acutely and chronically compared with medical therapy alone [15,16,17]. Weight loss is considered the primary factor in improving BP after SG, and it is thought that reducing visceral fat helps suppress sympathetic nervous system overactivity and systemic inflammation [18]. Interestingly, recent research has suggested that small-intestinal hormones such as glucagon-like peptide-1 (GLP-1), peptide YY (PYY) [19,20] and cholecystokinin (CCK) [21] may also play a role in the antihypertensive effect of SG. However, the detailed mechanisms are not yet fully understood, and few studies have investigated these mechanisms in detail.

In this study, we aimed to confirm the antihypertensive effect of SG and to investigate in detail the mechanisms underlying the antihypertensive effects through small-intestinal hormones independently of weight loss.

## 2. Materials and Methods

### 2.1. Animals

Eight-week-old male Sprague–Dawley rats (260–310 g; Jackson Laboratory Japan, Inc., Yokohama, Japan) were housed under a 12-h light/dark cycle (lights on 08:00–20:00) in a temperature-controlled room maintained at 23 ± 1 °C with 50 ± 10% relative humidity. Rats had ad libitum access to a standard diet (Oriental Yeast Co., Tokyo, Japan) and tap water. Rats were assigned to groups based on baseline body weight to ensure similar mean weights across groups. The experimental unit was the individual rat for all analyses. All experiments were approved by the Oita University Animal Experimentation Committee and conformed to the Oita University’s Guidelines for Animal Experimentation.

### 2.2. Experimental Protocol

**Figure 1:**
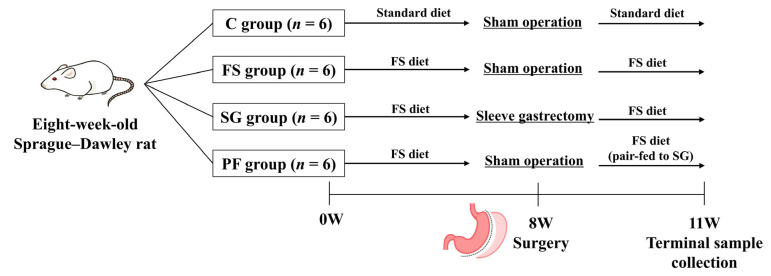
Experimental design and timeline (Experiment 1). Rats were assigned to the C, FS, SG, and PF groups (*n* = 6 per group). Surgery was performed after 8 weeks of housing, and terminal samples were collected 3 weeks postoperatively. C, rats fed a standard diet and underwent a sham-op; FS, rats fed a high-fat, high-salt diet and underwent a sham-op; SG, rats fed a high-fat, high-salt diet and underwent sleeve gastrectomy; PF, rats pair-fed the same amount of a high-fat, high-salt diet as the SG group and underwent a sham-op.

Experiment 1: Eight-week-old Sprague–Dawley rats were assigned to one of four groups to distinguish the effects of diet, surgery, and intake (*n* = 6 per group). Rats in group C were fed a standard diet for 8 weeks, underwent a sham operation (sham-op), and then continued on a standard diet for 3 weeks. The remaining rats were fed a high-fat, high-salt diet (FS diet: 60% kcal from fat, 20% from carbohydrate, 20% from protein, with 5% [*w*/*w*] NaCl; Research Diets, New Brunswick, NJ, USA) for 8 weeks and then divided into three groups: FS, SG, and PF. We used a high-salt diet to enhance sodium-handling signals and better detect the weight-independent antihypertensive effects of SG. Rats in the FS group underwent a sham-op and were fed an FS diet for 3 weeks. Rats in the SG group underwent SG and were fed an FS diet for 3 weeks. Rats in the PF group underwent a sham-op and were pair-fed the same amount of FS diet consumed by the SG group for 3 weeks. BP measurements and 24-h urine collections were obtained 3 weeks after surgery. Blood and tissue samples were collected after an 8-h fast. An overview of the experimental design is provided in Figure 1.

Experiment 2: Four groups of Sprague–Dawley rats (*n* = 2 per group) were fed either a standard diet or an FS diet and underwent either a sham-op or SG, as described in Experiment 1. The jejunum and ileum were collected and analyzed by RNA sequencing (RNA-seq).

**Figure 2:**
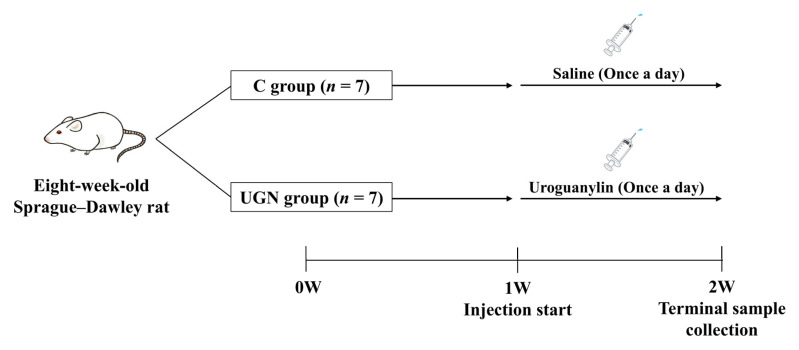
Experimental design and timeline (Experiment 3). Rats were assigned to the C and UGN groups (*n* = 7 per group). After 1 week of housing, uroguanylin or saline was administered once daily for 7 days. Terminal samples were collected on day 7. C, rats administered saline; UGN, rats administered uroguanylin.

Experiment 3: Eight-week-old Sprague–Dawley rats were assigned to two groups and maintained on a standard diet (*n* = 7 per group). Rats in the C group received daily intraperitoneal injections of saline for 1 week. Rats in the uroguanylin (UGN) group received daily intraperitoneal injections of UGN (100 nmol/kg in saline) for 1 week. BP was measured and 6-h urine was collected on days 1 and 7 of treatment. Samples were collected 2 h after administration. An overview of the experimental design is provided in Figure 2.

Eligibility criteria were defined before study initiation. All experiments used eight-week-old male Sprague–Dawley rats weighing 260–310 g. Exclusion criteria were limited to humane termination based on institutional policy and severe postoperative complications. No animals were excluded after study initiation. Blinding was not feasible due to surgical and operational constraints. To minimize bias, measurement conditions were standardized, assessment sequences and cage placements were alternated, and procedures, equipment, and analytical methods were kept uniform.

### 2.3. Sample Size and Power

The minimum detectable effect (MDE) for the primary endpoint of systolic blood pressure (SBP) was calculated using a sensitivity power analysis. With a two-sided significance level of α = 0.05 and power of 80%, the MDEs were 15.7 mmHg for Experiment 1 and 6.0 mmHg for Experiment 3. Based on these results, we concluded that Experiments 1 and 3 had sufficient sample sizes to detect differences in SBP between groups. Experiment 2 was conducted with the minimum necessary number of animals (*n* = 2 per group) as it was intended solely for exploratory screening via RNA-seq. Although the small sample size may limit statistical power, the reproducibility of proteins encoded by genes identified by RNA-seq was confirmed in Experiment 1 (*n* = 6 per group), supporting their biological relevance.

### 2.4. Animal Care Monitoring

In this study, all surgical procedures were performed under general anesthesia using a mixture of medetomidine (0.15 mg/kg), midazolam (2 mg/kg), and butorphanol (2.5 mg/kg) via intraperitoneal injection. All animals were carefully monitored postoperatively. Humane endpoints were defined to include weight loss of more than 20%, severe lethargy, inability to eat or drink, and uncontrolled bleeding. No adverse events were observed during the study period, and no animals were euthanized for meeting a humane endpoint.

### 2.5. SG

The rats were fasted for 20 h prior to surgery, after which they were anesthetized. The surgery was performed under aseptic conditions in the animal facility’s surgical suite. The SG procedure has been described previously [22,23]. Briefly, an incision was made in the midline of the upper abdomen, and the terminal esophagus, stomach, and initial duodenum were dissected. The greater curvature was incised from the antrum to the fundus of the stomach across the forestomach and the glandular stomach, and approximately 90% of the forestomach and 70% of the glandular stomach were resected. The divided stomach was closed using 4–0 nylon sutures. In the sham-op, the abdomen was opened, the stomach was lifted, and returned to the abdominal cavity. The sham-op was used as a control to account for the effects of laparotomy and anesthesia.

### 2.6. Reagent

Uroguanylin (rat) peptide was purchased from a commercial supplier (AnaSpec, Louisville, KY, USA) and dissolved in saline for use. The dose was selected based on prior reports to achieve detectable effects and a safety margin.

### 2.7. BP Measurement

SBP was recorded in conscious, resting animals using tail-cuff plethysmography (BP-98A; Softron, Tokyo, Japan). This method is noninvasive and allows for repeated measurements. The animals were placed in a plastic holder at 37 °C for 10 min to dilate the tail artery. Measurements were performed between 09:00 and 12:00, and the average was calculated from at least three readings obtained after each animal had acclimatized to the environment.

### 2.8. Protocols for Blood, Urine, and Tissue Collection and Storage

Once all rats were anesthetized, blood was collected from the right ventricle of the heart. These samples were then transferred to polypropylene tubes containing ethylenediaminetetraacetic acid (1 mg/mL, Sigma Aldrich, St. Louis, MO, USA) at 0 °C. Samples were then centrifuged at 2000× *g* for 15 min at 4 °C. The plasma was immediately frozen and stored at −80 °C until analysis. Urine was collected using metabolic cages and stored at −80 °C prior to analysis. Finally, the rats were exsanguinated following transcardial perfusion with 100 mL of saline. The jejunum, ileum, and kidney were removed and immediately frozen before being stored at −80 °C until analysis.

### 2.9. RNA Sequencing

Total RNA was isolated from the jejunum and ileum of each rat using TRIzol reagent (Thermo Fisher Scientific, Tokyo, Japan) and purified using the SV Total RNA Isolation System (Promega Corporation, Madison, WI, USA), according to the manufacturer’s instructions. RNA samples were quantified using an ND-1000 spectrophotometer (NanoDrop Technologies, Wilmington, DE, USA), and their quality was confirmed using a TapeStation (Agilent Technologies, Santa Clara, CA, USA). Sequencing libraries were prepared from 200 ng of total RNA using the MGIEasy rRNA Depletion Kit and MGIEasy RNA Directional Library Prep Set (MGI Tech Co., Shenzhen, China), according to the manufacturer’s instructions. The libraries were sequenced using a paired-end 150 nt strategy on the DNBSEQ-G400 FAST Sequencer (MGI Tech Co., Shenzhen, China). All sequencing reads were trimmed of low-quality bases and adapters using Trimmomatic software (version 0.38; Usadel Lab, RWTH Aachen University, Aachen, Germany) [24]. Raw counts for each gene in each sample were estimated using RSEM (version 1.3.0; University of Wisconsin–Madison, Madison, WI, USA) and Bowtie 2 (Ben Langmead Lab, Johns Hopkins University, Baltimore, MD, USA) [25,26]. Differentially expressed genes (DEGs) were identified using the edgeR program (Bioconductor project, Walter and Eliza Hall Institute of Medical Research, Melbourne, Australia) [27]. Normalized counts per million (CPM) values, log fold-changes (logFC), false discovery rate (FDR), and *p* values were calculated from the raw counts at the gene level.

### 2.10. Western Blot Analysis

Frozen tissue samples were homogenized in radioimmunoprecipitation assay buffer, then centrifuged, and boiled. The total protein concentration of each tissue sample was quantified using the Bradford assay, and 8 μg of total protein per sample was loaded onto a 4–20% SDS polyacrylamide gel. Proteins were separated using electrophoresis and transferred onto polyvinylidene fluoride membranes (Bio-Rad Laboratories, Richmond, CA, USA). The membranes were incubated for 1 h with primary antibodies against prouroguanylin (proUGN) (Santa Cruz Biotechnology, Santa Cruz, CA, USA), sodium/hydrogen ion exchanger type 3 (NHE3) (Sigma-Aldrich, Burlington, MA, USA), NHE3 phosphorylated at Ser552 (pS552-NHE3) (Novus Biologicals, Centennial, CO, USA), glyceraldehyde-3-phosphate dehydrogenase (GAPDH) (Abcam, Cambridge, UK), and β-actin (Santa Cruz Biotechnology, Santa Cruz, CA, USA). pS552-NHE3 has been reported to be an inactive form of NHE3 in proximal tubule cells [28]. Proteins were detected using enhanced chemiluminescence (Amersham Life Science, Buckinghamshire, UK) and quantified using ImageJ (version 1.54p; National Institutes of Health, Bethesda, MD, USA).

### 2.11. Immunohistochemistry

Kidney samples were fixed in buffered 4% paraformaldehyde, embedded in paraffin, sectioned, and subsequently deparaffinized in xylene. The tissues were then washed three times with phosphate-buffered saline and incubated for 1 h in 0.3% H_2_O_2_ to quench endogenous peroxidase activity. To stain for pS552-NHE3, 2 µm-thick kidney sections were incubated overnight at 4 °C with a mouse anti-rat pS552-NHE3 antibody (diluted 1:300; Novus Biologicals, Centennial, CO, USA). This was followed by incubation with biotin-conjugated horse anti-mouse immunoglobulin G (IgG) antibody (ABC reagent; Vector Laboratories, Burlingame, CA, USA). Visualization was performed using diaminobenzidine (DAB) substrate.

### 2.12. Enzyme-Linked Immunosorbent Assay (ELISA)

Plasma proUGN levels were measured using the rat guanylate cyclase activator 2B ELISA kit (Cusabio, Houston, TX, USA).

### 2.13. Measurement of Urinary Sodium

Urinary sodium concentrations were measured using ion-selective electrodes. Urinary sodium excretion was calculated as urinary sodium concentration (mmol/L) × urine volume (L).

### 2.14. Statistical Analysis

For each comparison, we reported the mean difference (MD) with a 95% confidence interval (CI), along with *p* values. Data were displayed as mean ± SEM. Statistical significance was defined as two-sided *p* < 0.05. Normality was satisfied for all analyses; therefore, prespecified parametric methods were applied. Homogeneity of variances was evaluated (Levene’s test); when this assumption was met, differences among multiple groups were analyzed using one-way analysis of variance (ANOVA) followed by Tukey’s post hoc test, whereas when variances were unequal, the Games–Howell post hoc test was applied. Data analysis was performed using IBM SPSS Statistics, version 29 (IBM Corporation, Armonk, NY, USA).

## 3. Results

### 3.1. Changes in Body Weight, Food Intake, BP, and Urinary Sodium Excretion

**Figure 3:**
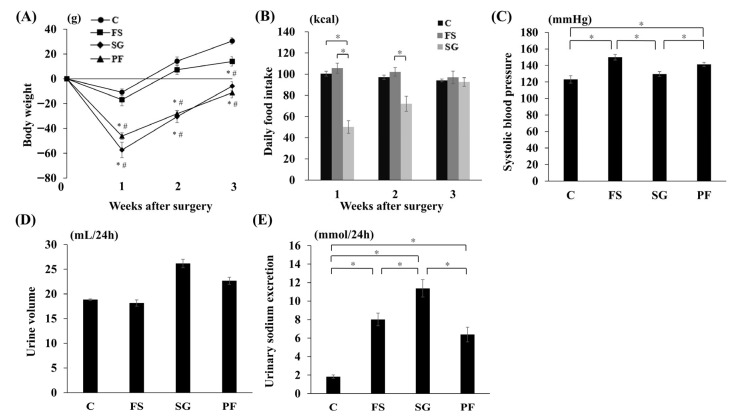
Changes in parameters after SG. Changes in body weight (**A**), food intake (**B**), systolic blood pressure (SG vs. FS: MD, −22.7 mmHg; 95% CI, −34.7 to −10.6; *p* < 0.001; SG vs. PF: MD, −16.3 mmHg; 95% CI, −28.4 to −4.3; *p* = 0.006) (**C**), urine volume (**D**), and urinary sodium excretion (SG vs. C: MD, 9.5 mmol/24 h; 95% CI, 6.7 to 12.3; *p* < 0.001; SG vs. FS: MD, 3.7 mmol/24 h; 95% CI, 0.6 to 6.2; *p* = 0.015; SG vs. PF: MD, 3.9 mmol/24 h; 95% CI, 1.1 to 6.7; *p* = 0.004) (**E**) after SG. Data are mean values ± SEM. * *p* < 0.05 versus the C group; ^#^ *p* < 0.05 versus the FS group. C, rats fed a standard diet and subjected to a sham-op; FS, rats fed a high-fat and high-salt diet and subjected to a sham-op; SG, rats fed a high-fat and high-salt diet and subjected to sleeve gastrectomy; PF, rats fed the same amount of a high-fat and high-salt diet as the SG group and subjected to a sham-op.

In Experiment 1, at 3 weeks post-surgery, body weight was lower in the SG and PF groups than in the C and FS groups, with no differences between the SG and PF groups or between the C and FS groups (Figure 3A). Postoperative daily food intake was transiently reduced in the SG group compared with the C and FS groups (Figure 3B). SBP at 3 weeks post-surgery was lower in the SG group than in the FS and PF groups (Figure 3C). At 3 weeks post-surgery, no significant differences in 24-h urine volume were observed among the four groups (Figure 3D). In contrast, 24-h urinary sodium excretion was higher in the SG group than in the other groups (Figure 3E).

### 3.2. RNA Sequencing of the Jejunum and Ileum

**Figure 4:**
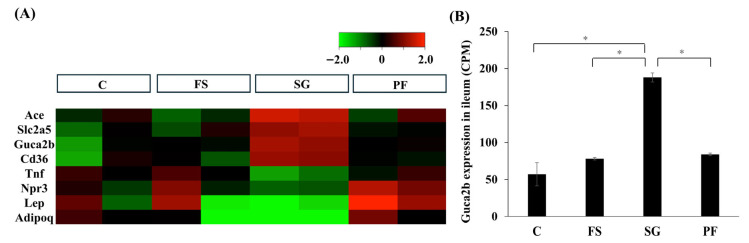
RNA sequencing of the ileum after SG. In the ileum, 13,420 genes were detected, of which 634 showed significant (*p* < 0.05, FDR < 0.05) and large changes in expression (logFC > 1 or logFC < −1) in the SG group compared with the FS and PF groups. Eight genes were classified into the “regulation of blood pressure” category (**A**). Guca2b expression was significantly higher in the SG group (SG vs. C: MD, 131.5; 95% CI, 29.3 to 233.7; *p* < 0.001; SG vs. FS: MD, 110.4; 95% CI, 72.0 to 148.8; *p* < 0.001; SG vs. PF: MD, 104.2; 95% CI, 65.7 to 142.7; *p* < 0.001) (**B**). Data are mean values ± SEM. * *p* < 0.05. C, rats fed a standard diet and subjected to a sham-op; FS, rats fed a high-fat and high-salt diet and subjected to a sham-op; SG, rats fed a high-fat and high-salt diet and subjected to sleeve gastrectomy; PF, rats fed the same amount of a high-fat and high-salt diet as the SG group and subjected to a sham-op; CPM, counts per million.

In Experiment 2, RNA-seq was performed on samples from the jejunum and ileum. A total of 13,274 genes were identified in the jejunum, but none of these genes belonged to the “regulation of blood pressure” category (GO:0008217). In the ileum, eight genes were categorized as “regulation of blood pressure” (GO:0008217) (Figure 4A). Notably, Guca2b expression was significantly higher in the SG group than in the other groups (Figure 4B).

### 3.3. Changes in the Expression of proUGN and pS552-NHE3 Due to SG

**Figure 5:**
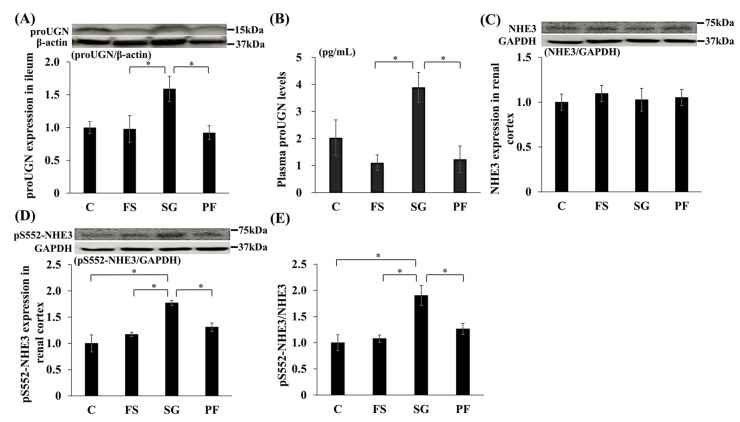
Changes in the expression of proUGN, NHE3 and pS552-NHE3 after SG. Changes in proUGN protein expression in the ileum (SG vs. FS: MD, 0.66; 95% CI, 0.01 to 1.31; *p* = 0.046; SG vs. PF: MD, 0.72; 95% CI, 0.07 to 1.37; *p* = 0.028) (**A**) and plasma (SG vs. FS: MD, 2.79 pg/mL; 95% CI, 0.36 to 5.23; *p* = 0.021; SG vs. PF: MD, 2.66 pg/mL; 95% CI, 0.22 to 5.10; *p* = 0.029) (**B**), NHE3 (**C**) and pS552-NHE3 (SG vs. C: MD, 0.77; 95% CI, 0.36 to 1.18; *p* < 0.001; SG vs. FS: MD, 0.60; 95% CI, 0.19 to 1.01; *p* = 0.003; SG vs. PF: MD, 0.46; 95% CI, 0.047 to 0.88; *p* = 0.026) (**D**) protein expression in the renal cortex, and the relative expression of pS552-NHE3 to NHE3 (SG vs. C: MD, 0.90; 95% CI, 0.31 to 1.50; *p* = 0.020; SG vs. FS: MD, 0.82; 95% CI, 0.23 to 1.42; *p* = 0.005; SG vs. PF: MD, 0.64; 95% CI, 0.04 to 1.24; *p* = 0.033) (**E**) after SG. Data are mean values ± SEM. * *p* < 0.05. C, rats fed a standard diet and subjected to a sham-op; FS, rats fed a high-fat and high-salt diet and subjected to a sham-op; SG, rats fed a high-fat and high-salt diet and subjected to sleeve gastrectomy; PF, rats fed the same amount of a high-fat and high-salt diet as the SG group and subjected to a sham-op; GAPDH, glyceraldehyde-3-phosphate dehydrogenase; NHE3, sodium/hydrogen ion exchanger type 3; proUGN, prouroguanylin; pS552-NHE3, NHE3 phosphorylated at Ser552.

**Figure 6:**
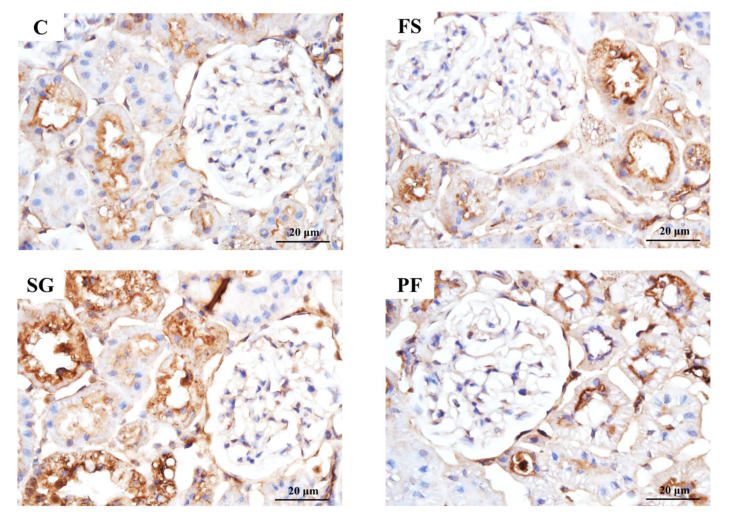
pS552-NHE3 immunostaining of the renal cortex after SG. Histology showed glomerular hypertrophy in the FS group compared with the C group, but no marked differences between the SG and PF groups. pS552-NHE3 expression in the proximal tubules of the SG group was higher than that in all other groups.

In Experiment 1, proUGN protein expression in the ileum and plasma was higher in the SG group than in the FS and PF groups (Figure 5A,B). In the renal cortex, NHE3 expression did not differ among groups, whereas levels of pS552-NHE3, which is an inactive form of NHE3, and the pS552-NHE3/NHE3 ratio were higher in the SG group than in the other groups (Figure 5C–E). Immunohistochemistry revealed increased pS552-NHE3 expression in the proximal tubules of the SG group (Figure 6).

### 3.4. Effects of UGN on BP, Urinary Sodium Excretion and Renal pS552-NHE3

**Figure 7:**
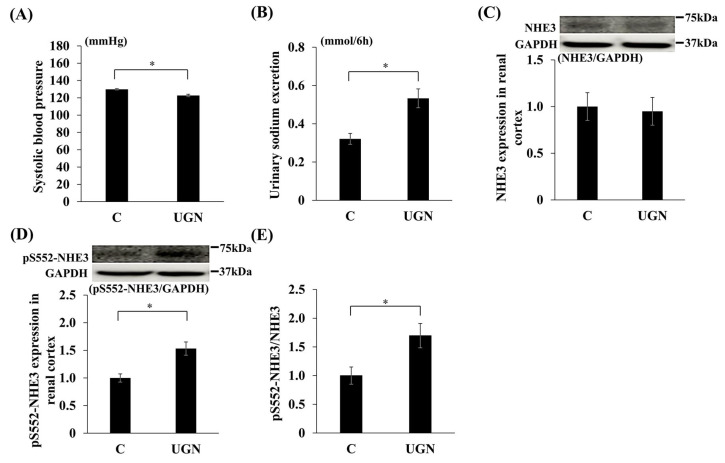
Changes in parameters after the administration of UGN. Changes in systolic blood pressure on day 7 (UGN vs. C on day 1: MD, −7.7 mmHg; 95% CI, −13.0 to −2.4; *p* = 0.008; on day 7: MD, −7.0 mmHg; 95% CI, −11.2 to −2.8; *p* = 0.004) (**A**), urinary sodium excretion on day 7 (UGN vs. C on day 1: MD, 0.14 mmol/6 h; 95% CI, 0.014 to 0.26; *p* = 0.032; on day 7: MD, 0.21 mmol/6 h; 95% CI, 0.08 to 0.34; *p* = 0.005) (**B**), NHE3 (**C**) and pS552-NHE3 (UGN vs. C: MD, 0.53; 95% CI, 0.20 to 0.87; *p* = 0.002) (**D**) protein expression in the renal cortex, and the relative expression of pS552-NHE3 to NHE3 (UGN vs. C: MD, 0.70; 95% CI, 0.09 to 1.31; *p* = 0.027) (**E**). Data are mean values ± SEM. * *p* < 0.05. C, rats administered saline solution; UGN, rats administered uroguanylin solution; GAPDH, glyceraldehyde-3-phosphate dehydrogenase; NHE3, sodium/hydrogen ion exchanger type 3; proUGN, prouroguanylin; pS552-NHE3, NHE3 phosphorylated at Ser552.

**Figure 8:**
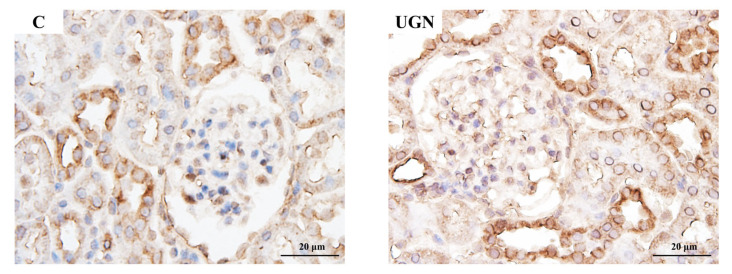
pS552-NHE3 immunostaining of the renal cortex after the administration of UGN. The expression of pS552-NHE3 in the C and UGN groups. pS552-NHE3 expression in the proximal tubules was higher in the UGN group than in the C group.

In Experiment 3, intraperitoneal administration of UGN, which is converted from proUGN, for 1 week reduced SBP and increased urinary sodium excretion compared with saline-treated controls (Figure 7A,B). UGN did not change NHE3 levels in the renal cortex but increased pS552-NHE3 levels and the pS552-NHE3/NHE3 ratio (Figure 7C–E). Consistent with these findings, pS552-NHE3 staining in proximal tubules was enhanced in the UGN group (Figure 8).

## 4. Discussion

In this study, we investigated the antihypertensive effects of SG in obese hypertensive rats. SG significantly reduced BP and increased urinary sodium excretion. RNA-seq of the small intestine revealed that Guca2b gene expression, which is associated with BP and natriuresis, was significantly higher in the SG group. The proUGN protein, which is encoded by the Guca2b gene, was significantly higher in the ileum and plasma after SG. Additionally, pS552-NHE3 expression was significantly increased in the renal cortex. Further experiments showed that administration of exogenous UGN produced effects similar to those of SG, including reduced BP, increased urinary sodium excretion, and increased pS552-NHE3 in the renal cortex.

### 4.1. Factors Associated with the Antihypertensive Effect of SG

SG has been shown to significantly improve hypertension in patients with severe obesity [18]. While weight loss is the most important factor in this mechanism, BP significantly decreases even in the early postoperative stage, when weight loss is limited [29]. Furthermore, rats that underwent SG had significantly lower BP than rats in the PF group, which achieved similar weight loss [30]. These findings suggest that the antihypertensive effect of SG cannot be explained by weight loss alone, and that multiple potential mechanisms exist. Among the potential mechanisms of SG’s antihypertensive effects, the involvement of small-intestinal hormones has been reported. Yoshida et al. reported that SG increased the secretion of the small-intestinal hormone CCK, which inhibits the renal sympathetic nervous system, thereby suppressing the renin-angiotensin-aldosterone system in the kidney, increasing urinary sodium excretion, and reducing BP [21]. Furthermore, SG has been reported to increase the secretion of small-intestinal hormones, such as GLP-1 and PYY, which promote natriuresis and inhibit the sympathetic nervous system [19,20]. However, the exact mechanism through which SG exerts its antihypertensive effects remains unclear. In this study, the SG group exhibited significantly lower BP despite having equivalent weight loss and salt intake than the PF group at 3 weeks post-surgery. This finding suggests the presence of antihypertensive mechanisms other than weight loss and salt intake. Furthermore, the SG group exhibited increased urinary sodium excretion compared with the PF group, indicating that this may contribute to the antihypertensive effect of SG.

In our analysis of BP and body weight in the FS and C groups, the FS group exhibited higher BP than the C group at 3 weeks post-surgery, whereas postoperative weight gain did not exceed that of the C group. We hypothesize that pre-existing obesity and high-salt intake in the FS group may have enhanced the inflammatory response and increased energy expenditure after surgery, thereby limiting further weight gain.

### 4.2. SG and RNA Sequencing

RNA-seq is a technique that uses next-generation sequencers to comprehensively sequence the cDNA prepared from intracellular RNA. This has contributed significantly to the elucidation of complex gene expression and transcriptional regulatory mechanisms [31]. Recent studies have applied RNA-seq to the pancreas [32], adipose tissue [33] and liver [34] after SG, identifying factors that contribute to improving metabolic disorders such as diabetes and fatty liver disease. These findings demonstrate that RNA-seq is an extremely useful tool for elucidating the mechanisms underlying the improvement of metabolic disorders after SG. However, no studies have yet investigated the antihypertensive mechanisms of SG using RNA-seq. In this study, RNA-seq was performed on the jejunum and ileum to explore antihypertensive mechanisms other than those induced by weight loss after SG. In the SG group, Guca2b, which encodes proUGN, was identified as a gene that was significantly altered and associated with the “regulation of blood pressure” category. Additionally, Guca2b was the only gene identified as associated with the “renal sodium absorption” category. Based on these findings, we hypothesized that proUGN, encoded by Guca2b, plays a key role in the antihypertensive and sodium excretion-enhancing effects of SG. Other genes previously associated with BP or natriuresis, such as CCK, GLP-1, and PYY, showed no significant changes in expression in the SG group.

### 4.3. Association Between proUGN and the Antihypertensive Effects of SG

**Figure 9:**
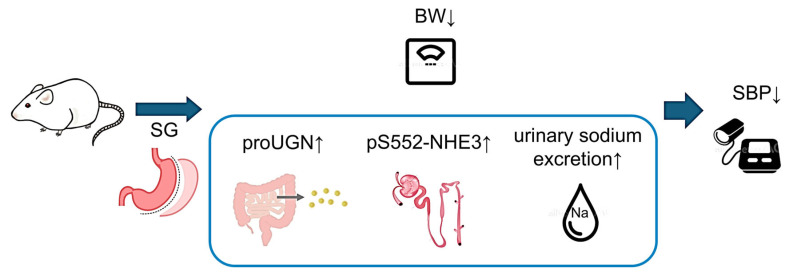
Mechanism of blood pressure reduction following SG. SG increased proUGN secretion from the small intestine, increasing its circulating concentration. The enhanced proUGN increased pS552-NHE3 in proximal tubules, promoted natriuresis, and lowered blood pressure. BW, body weight; proUGN, prouroguanylin; pS552-NHE3, NHE3 phosphorylated at Ser552; SBP, systolic blood pressure; SG, sleeve gastrectomy.

proUGN is released into the blood from enterochromaffin cells in the small intestine and is converted into active UGN in the kidneys [35]. UGN then binds to guanylate cyclase C (GC-C) receptors in the proximal tubules, increasing the levels of cyclic guanosine monophosphate (cGMP) and cyclic adenosine monophosphate (cAMP). Subsequently, cGMP activates cGMP-dependent protein kinase, which in turn activates cAMP-dependent protein kinase. These kinases primarily phosphorylate Ser552 at the C-terminal end of NHE3, converting it into the inactive form, pS552-NHE3. Therefore, an increase in pS552-NHE3 expression indicates inhibition of NHE3 activity. NHE3 is responsible for reabsorbing sodium in the proximal tubules; therefore, inhibition of its activity by increased pS552-NHE3 levels promotes sodium excretion [36]. While it has been reported that SG increases proUGN blood levels in humans and rats [37], no studies have compared SG with PF. In the present study, significantly higher proUGN levels were observed in the ileum and plasma of the SG group than in the FS and PF groups. Additionally, a significant increase in pS552-NHE3 expression was observed in the renal cortex. As proUGN is secreted by the digestive tract in response to food intake, particularly salt intake [38], the reduction in gastric volume caused by SG may have resulted in faster transit of food to the small intestine. This may have stimulated or altered intestinal microbiota, thereby increasing proUGN secretion. However, the detailed mechanisms underlying the SG-induced increase in proUGN secretion remain unclear, and further studies are necessary. In summary, SG increased the secretion of proUGN from the small intestine, leading to an increase in its blood concentration. This resulted in increased pS552-NHE3 expression in the renal cortex, increased urinary sodium excretion, and reduced BP (Figure 9).

### 4.4. Changes in BP After the Administration of UGN

In an additional experiment, UGN was administered to rats. Previous studies have reported that UGN administration increases sodium excretion in rats [39] and that although UGN administration to proximal tubule cells does not alter NHE3, the expression of pS552-NHE3 relative to NHE3 significantly increases [36]. In another study, changes in BP after UGN administration showed a decreasing trend, although no significant changes were observed [40]. This may have been due to the low dosage or short administration period. In UGN-knockout mice, increased BP [41] and reduced urinary sodium excretion [42] have been reported, leading to the expectation of a hypotensive effect of UGN. In this study, UGN administration for 1 week resulted in increased pS552-NHE3 in the renal cortex, increased urinary sodium excretion, and decreased BP without body weight reduction. These findings support the hypotensive effect of SG and the involvement of UGN.

## 5. Conclusions

This study identified proUGN as an independent factor that contributes to the antihypertensive effect of SG, regardless of weight loss. This finding might have important clinical implications, as targeting the antihypertensive pathway mediated by proUGN could lead to new therapies for obesity-related hypertension. Therefore, these findings need to be validated in large-scale studies to explore their clinical applications.

## 6. Limitations

This study had several limitations. First, the study was confined to experiments involving rats; therefore, the results may not be applicable to humans. Second, the tail-cuff method was used to measure BP. While this method is simple and noninvasive, the environment, restraint of the rat, and attachment of the cuff may have affected BP values. Third, the follow-up period was limited to 3 weeks after surgery. It remains unclear whether the observed effects will persist in the long term. Fourth, we did not thoroughly assess blood volume, which may influence BP. It would be desirable to evaluate this in future studies. Fifth, body weight had returned to baseline by 3 weeks postoperatively, reflecting ongoing growth and the relatively modest extent of gastric resection. Thus, translational alignment with clinical practice is limited. Finally, no specific antibodies against UGN are commercially available. Therefore, only proUGN expression could be evaluated. Future studies are required to evaluate UGN expression when assays become available.

## Data Availability

The data that support the findings of this study are available from the corresponding author upon reasonable request. The RNA-seq data generated in this study have been deposited in the European Nucleotide Archive (ENA) and are available under accession number [ENA: PRJEB102361].
